# Effects of Minoxidil Gel on Burn Wound Healing in Rats 

**Published:** 2014

**Authors:** Payam Khazaeli, Mohammad Karamouzian, Shohreh Rohani, Behnam Sadeghirad, Nima Ghalekhani

**Affiliations:** a School of Pharmacy and Pharmaceutical Research Center, Kerman University of Medical Sciences, Kerman, Iran.; b Regional Knowledge Hub, and WHO Collaborating Centre for HIV Surveillance, Institute for Futures Studies in Health, Kerman University of Medical Sciences, Kerman, Iran.; c School of Pharmacy, Kerman University of Medical Sciences, Kerman, Iran.; d Neuroscience Research Center, Institute of Neuropharmacology, Kerman University of Medical Sciences, Kerman, Iran*. *; e Research Center for Health Services Management, Institute for Futures Studies in Health, Kerman University of Medical Sciences, Kerman, Iran.

**Keywords:** Minoxidil, Topical gel, Angiogenesis, Second-degree burn, Wound healing

## Abstract

Minoxidil has been reported to inhibit *in*-*vitro* fibroblast proliferation and lysyl hydroxylase activity, a key enzyme in collagen biosynthesis. These *in-vitro* effects proposed minoxidil to be a potential antifibrotic agent. The present study aimed to investigate the effects of minoxidil gel on wound healing procedure in a second-degree burn model in rats.

Wistar rats were anesthetized and a second-degree burn was induced on the back of Wistar rats using a heated 2 cm diameter metal plate. Experimental groups received 2% or 5% topical minoxidil gel, dexpanthenol or sliver sulfadiazine. Histological parameters including collagen content, angiogenesis, number of preserved follicles and necrosis along with tensile strength of burn wound area were assessed on days 3, 7, 14 and 21 post-injury.Microscopic evaluation of specimens collected from sample animals were consistent and showed a second-degree burn. Main histological findings regarding minoxidil topical usage showed that collagen content and tensile strength of burned area did not differ between groups. However, minoxidil increased the number and diameter of blood vessels significantly compared with other groups.Although minoxidil improved the process of wound-healing, our results did not support the proposed idea of its usage as an antifibrotic agent. However, to reject its possible effects as an antifibrotic agent, more objective animal models should be developed and studied.

## Introduction

Minoxidil (MOX) is a pyrimidine derivative initially used as a potent antihypertensive agent which is a vasorelaxant agent that opening ATP-sensitive K channel (1, 2). Minoxidil induces hypertrichosis as a common side effect (2, 3), which result in development of topical formulations for treatment of male pattern baldness. Almost three decades ago, Cohen et al showed that MOX could have direct effects on the proliferation and morphology of epidermal cells (4). A few years later, Murad and Pinnel demonstrated that MOX inhibits fibroblast proliferation without causing cytotoxicity (5).

In addition to inhibitory effects of MOX on fibroblasts proliferation and activity, it showed inhibitory effects on keratinocytes proliferation and delays keratinocytes differentiation and senescence *in-vitro *(6). It is reported that, MOX can up-regulates the expression of vascular endothelial growth factor (VEGF), which has central role in formation of new blood cells and promoting angiogenesis, and affects eicosanoid production, that have anti-inflammatory properties and stimulate collagen synthesis, and improve blood flow to the skin(3,7). Several studies reported that MOX inhibited lysyl hydroxylase (LH) and dramatically reduced the level of LH activity in dermal fibroblasts (4,8-16). LH catalyzes the hydroxylation of lysine residues in peptide linkages and it is a key post-translational modifying enzyme in collagen biosynthesis (8, 14, 17).

MOX effects on fibroblast proliferation and metabolism, and its interference with crucial steps in collagen biosynthesis, revealed its potential ability to inhibit the contractile properties of the cells responsible for wound contraction. Hence, it is suggested that it might be an effective topical treatment for fibroses, hypertrophic scar, or keloids, where collagen accumulates (5, 18). Many compounds with promising activities against collagen synthesis or fibroblast proliferation are disqualified from use because of their toxicity, but MOX appears to fulfill all the criteria;it modulates a range of fibroblast activities without toxic effects (18), inhibits collagen synthesis and cross-linking (16), and collagen lattices contraction (19), and has influence on vascularization (7) and skin blood flow (2).

One of the most important wounds are burn wounds which is Susceptible to infection because of vascular necrotic tissue. Burn wound infection is main reason of morbidity in these patients (20, 21). 

 Scar formation in the process of burn wound healing involves three phases, which merge and overlap, the inflammatory phase, proliferative phase and remodeling phase (22-24). The main function of the first phase is to prepare the wound for repair. The second phase begins with the appearance of fibroblasts and its main functions are the restoration of collagen lattices and angiogenesis. The strength of the wound improves during this phase, and is proportional to the production of collagen (25, 26). The remodeling, begins after a few weeks, and may last years. During this stage, due to a dynamic balance between collagen synthesis and degradation wound strength increases with no actual change in collagen content (22-24). The final process of healing is contraction; it is the migration of the wound edges toward the center which results in reducing the wound size and fibroblasts are responsible for wound contraction (22-24).

Based on MOX properties and its effects on fibroblasts, collagen formation, and angiogenesis it is probable that a topical formulation of MOX may have influence on the processes involved in phase I and II of scar formation. Hence, in this study, we aimed to investigate the effects of MOX topical gel formulation on the healing process of a murine partial second-degree burn model and to compare MOX effects with two routine burn wound treatments (dexpanthenol ointment and silver sulfadiazine cream).

## Experimental

*Drugs*

To prepare 2% and 5% gel formulations, MOX powder (purchased from MERCK, Germany) was dissolved in appropriate amount of PEG 400. Then, boiling ethanol was added to the heated MOX solution while stirring. This solution was added to the carbomer containing mixture and stirred until being cooled. Silver sulfadiazine cream 1% (Sina-Daru Inc., Iran) and dexpanthenol 5% (Pars-Daru Inc., Iran) ointments were commercially purchased.

*Experimental protocol*

An experimental skin burn model was prepared as described below. Wistar albino rats were anaesthetized with an intraperitoneal injection of ketamine (60 mg kg^-1^) and xylazine HCL (8 mg kg^-1^). Mean age for test animals was 75 ± 15 days and their mean weight was 215 ± 21g. The skin of dorsum was shaved and thoroughly depilated. Burn injury was made as a contact burn with a round 2 cm diameter metal plate (105 ˚C, 5 seconds). An hour after burn injury, each animal received an appropriate amount of topical formulation with regards to the assigned group. Topical applications were repeated every day and continued until 12 h before sample collection. 

Animals were assigned randomly to either control or experimental groups. Experimental groups was further subdivided into five groups as follows: Group 1 received topical blank gel (formulated gel base without minoxidil), group 2 received topical dexpanthenol ointment as standard healing agent, group 3 received topical sliver sulfadiazine (SSD) ointment as standard burn treatment (27, 28), group 4 and 5 received 2% and 5% topical minoxidil gel (n > 5 rats per group per time point, total of 257 animals).

In order to collect specimens, the animals were euthanized at 3, 7, 14 and 21 days after inducing burn injury in a chamber filled with excessive amount of diethyl ether. These specimens were preserved in 10% formalin for light microscopic study. Animals in this study were treated strictly according to the supervision of the Ethics Committee of Kerman University of Medical Sciences.

*Histological assessments*

The parameters investigated included angiogenesis and their total diameter, number of preserved follicles, reticular fibers and collagen formation, the number of inflammatory cells, and necrosis (29, 30). From each sample, two slides were prepared and the related parameters were quantified in at least three randomly selected fields. All histological procedures were performed by two independent blind observers. 

Standard histological slices were stained using hematoxylin–eosin (blood vessels, necrosis, and inflammatory cells), Gomori silver stain (reticular fibers) and Van Gieson staining (collagen). The assessed parameters according to day of sacrifice were as follows: day3: total number and diameter of blood vessels, number of inflammatory cells and number of vital follicles; day 7, 14 and 21: as day 3 with percentage of reticular fibers and collagen (27, 28). 

Angiogenesis has been determined by measuring the number and sum of diameters of blood vessels. Two microscopic fields (mag. 6.3×) in the central part of the burned area were analyzed to determine this parameter (27, 28). Inflammatory cells have been counted in three visual fields in the middle portion of the burned area (mag. 40×) between the deepest layer of follicles and the muscular layer of the skin (27, 28). Necrosis has been scored using a modified Suzuki scale (mag. 25×) (31): Grade 1: necrosis within the epidermal layer; Grade 2: necrosis up to the deepest layer of hair follicles; Grade 3: necrosis exceeding the deepest layer of hair follicles; Grade 4: necrosis exceeding the muscular layer. The number of preserved follicles has been counted in three microscopic fields (mag. 25×) in the middle of the burned area and examining the deepest layer of hair follicles. Reticular fibers and collagen have been determined as percentages in two microscopic fields (mag. 40×) on each margin of the burned area between the deepest follicles and the muscular layer (27, 28).

*Tensile strength*

Tensile strength (the force required to open the healing skin) was used to measure the amount of healing (32, 33). The tensile strength of burned skin in control and treatment groups was measured on day 3 and day 7 post-injury using an industrial tensiometer (500 N, Bual, Germany). Strips of the burned wound were excised to compare changes in breaking strength as a function of time after wounding. The specimens were stretched between the machine clamps and the maximum force generated to torn specimens apart was measured (32, 33).

*Statistical analysis*

To compare tensile strength, number and diameter of blood vessels, number of preserved follicles and percentage of reticular fibers and collagen in different experimental groups, ANOVA test followed by Tukey or Scheffe post-hoc tests were used. In order to compare the number of inflammatory cells and necrosis among groups, Kruskal-Wallis test was used. Finally, linear regression model was used in order to assess the time trend of each parameter in different study groups. All data collected from experiments were analyzed using SPSS version 15.0.

## Results

There were no changes in food intake, water consumption and body weight of the animals subjected to burn injury. The wound lesions showed swelling immediately after the burn injury. Thereafter, the lesions became necrotic and were covered with a crust by day 7. The burn sizes did not differ between day 1 and day 7. On the other hand, the burn wounds decreased in size from day 7 to day 14. Besides, there was no significant difference between control and blank gel animals in any of histological assessment or breaking tensile strength.

**Figure 1:**
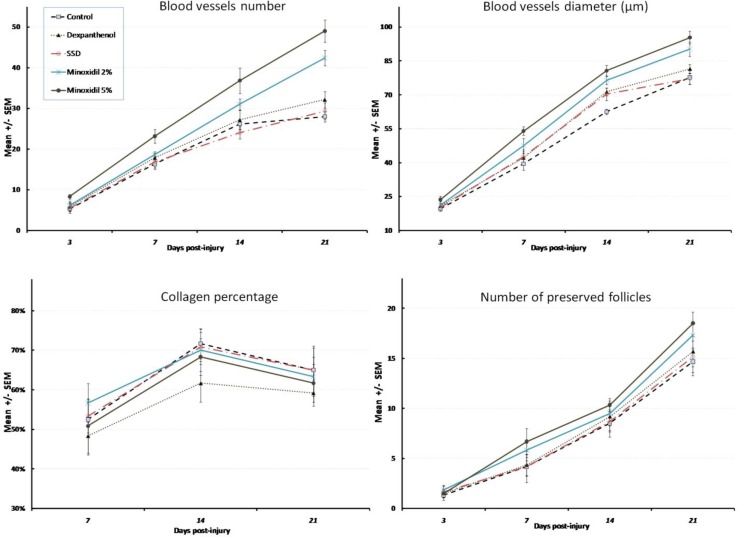
Histological assessments in different treatment groups during 21 days post-burn

There was a marked increase in blood vessel formation, and enhanced proliferation of hair follicles because of treatment with minoxidil gels post-burn (Figure 1). Total number (BVN) and diameter (BVD) of blood vessels showed no difference between different groups on day 3 (p = 0.202, F = 1.56 and p = 0.108, F = 1.99 for BVN and BVD respectively). However, minoxidil 5% gel formulation showed a significant increase in BVN and BVD compared with the control group after a week (p = 0.009 and p = 0.01 respectively). The difference between minoxidil 5% and dexpanthenol or SSD ointments showed a borderline significance (p = 0.066 and p = 0.051 for BVN and p = 0.069 and p = 0.053 for BVD).

On day 14^th ^post-burn, both minoxidil formulations showed a significant increased BVN compared to control (p = 0.007 and p = 0.001 for 2% and 5% formulations respectively). However, only 5% minoxidil gel increased BVD significantly (p = 0.006). While there was no significant difference between minoxidil formulations, the difference between minoxidil 5% gel and both dexpanthenol and SSD in total BVN was statistically significant (p = 0.015 and p = 0.004 respectively). A borderline significant difference was observed between minoxidil 5% and dexpanthenol or SSD in total BVD (p = 0.061 and p = 0.052 respectively).

**Figure 2:**
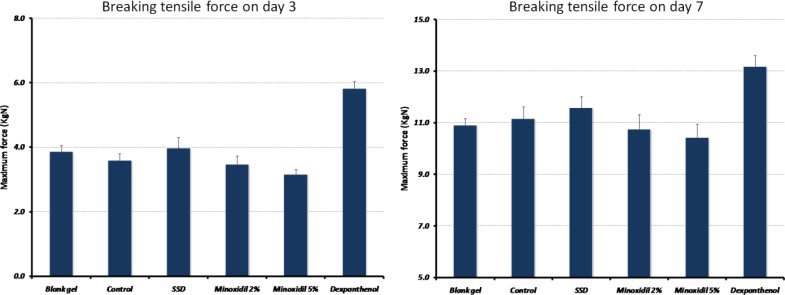
Tensiometry assessment. Breaking tensile force (Kg/N) in different treatment groups’ day 3 post-burn (left) and day 7 post-burn (right).

Three weeks after injury, minoxidil formulations showed still a significant higher amount of blood vessels formation (p = 0.009 and p = 0.033 for 2% and 5% formulations in BVD respectively and p < 0.001 and p = 0.001 for 2% and 5% formulations in BVN respectively). In spite of the significant difference between minoxidil gel formulations (2% and 5%) and SSD ointment in total BVD (p = 0.024 and 0.004 respectively) and BVN (p = 0.005 and p < 0.001 respectively), the difference between two minoxidil gel formulations was not statistically significant. Besides, the increase of BVN caused by both of the minoxidil gel formulations was more than dexpanthenol ointment, and their difference was statistically significant (p = 0.001 and p = 0.037 for 2% and 5% minoxidil gels). However, this difference was significant only for minoxidil 5% gel for BVD in comparison with dexpanthenol ointment (p = 0.014).

Evaluation of collagen and reticulin production during days post-injury showed that the difference between treatment groups with control or blank gel animals was not statistically significant. Reticular fibers or collagen percentage did not differ statistically between treatment groups either. Compared with control, only dexpanthenol ointment decreased the number of inflammatory cells during 21 days post-injury significantly (p = 0.002). Minoxidil formulations throughout the experiments increased the number of follicles compared with blank gel and control animals significantly (1.5 ± 0.7 increase in the number of follicles per day, p = 0.03 and 2.1 ± 0.7 increase in the number of follicles per day, p = 0.002 for 2% and 5% minoxidil gels). Less necrosis was observed in dexpanthenol animals during days post-injury compared with controls (p = 0.027), while other treated animals did not show any significant decreasing trend in necrosis score.

Comparing tensile strength in different groups, a significant difference was observed (p = 0.001, F = 16.0 and p = 0.003, F = 4.6 for day 3 and 7 post-injury respectively) (Figure 2). The results showed that 1.84 ± 0.51 KgN per day was added to the maximal stress until the wound disrupted (p = 0.001). Compared with the control group, only wounds treated with dexpanthenol demonstrated a significant increase in the amount of force needed for wound disruption (2.12 ± 0.3, p < 0.001).

## Discussion

To investigate the effects of minoxidil on wound healing, it was formulated for the first time in this study as a topical gel that could be readily used. Our results showed that neither 2%, nor 5% minoxidil gel altered collagen content of burn wounds throughout healing process compared to the control group animals. Besides, the tensile strength of burned area of the skin as an indicator of wound contraction was not different between minoxidil treated and control animals.

Burn injury is the first and the foremost cause of damage to the skin. Although the burned patient has many problems to face during the stages of recovery from a burn injury, the major and persisting problems for survivors are those associated with the problems of healing and the outcome of healing in terms of scarring. Problems associated with wound management, treatment and healing have always been important challenges for clinicians and investigators (34).

Wound contraction is the centripetal or concentric reduction in size of an open wound. It is a major component of second-intention wound healing, and the center for contraction is granulation tissue. The basic components of granulation tissue are fibroblasts, macrophages, capillaries, and collagen (35). Furthermore, the sources of cells involved in the healing of skin include fibroblasts, smooth muscle cells and endothelial cells in the dermis and associated structures (22, 36).

Minoxidil is a direct acting peripheral vasodilator recommended orally for treatment of severe hypertension (3, 37). In addition, it was unexpectedly found to stimulate hair growth. This major side effect of minoxidil led to its clinical use for treatment of common baldness (androgenic alopecia) and alopecia areata (38). Minoxidil was later reported to have direct effects on the proliferation and morphology of epidermal cells in concentrations comparable to those used clinically in treatment of alopecia (4). It has been proposed as a potential topical inhibitor of wound contraction and proliferative scaring (5-7, 18, 19, 39-41). Suggestions for this application are derived from *in-vitro* studies demonstrating that minoxidil inhibits fibroblast proliferation and activity (5, 18, 42-44).

Besides, Minoxidil is reported to depress lysyl hydroxylase activity, a key enzyme for collagen crosslinking, selectively in cultured human skin fibroblast and reduce collagen lattices (5, 11, 16, 19, 45). Minoxidil is also able to induce vasodilation, stimulate cutaneous blood flow and up-regulates the expression of VEGF, which is related to the formation of capillaries (2, 7).

In contrast to its stimulatory effects on epithelial cells, minoxidil has been shown to have a variety of inhibitory effects on fibroblasts *in-vitro*. These effects occur at concentrations much lower than 2% (approximately 100 mM) topical solution that is applied to intact skin to generate hair growth (3). In fact, at concentrations greater than 4 mM, minoxidil exhibits a significant cytotoxicity to the fibroblasts *in-vitro *(18, 45). Because this study involved direct topical application of minoxidil to target cells in burned wounds, 2% and 5% formulations were chosen. Besides, due to the high alcohol content of commercial topical solutions, minoxidil was formulated as a topical gel. Despite use of high minoxidil doses and the least irritating formulations, inhibitory effects were not demonstrated.

*In-vitro* culture is an incomplete method of studying wound dynamics because of the complexity of the *in-vivo* interactions between tissue tension, effector cells, extracellular matrix, humoral factors and locally derived growth factors (35, 46, 47). Reproducible animal models such as the one used in this study are useful tools to verify and validate the results of *in-vitro* study of therapeutic agents that may potentially alter wound healing process. Despite the abundance of the *in-vitro* data previously cited the multilevel inhibition of fibroblast metabolism and contraction of collagen lattices by minoxidil (5, 18, 19, 42, 45), this *in-vivo* study did not demonstrate comparable fibroblastic or collagen production inhibition considering the histological assessments and tensile strength.

If tensile strength can be assumed to be directly related to collagen crosslinking, the inhibition of lysyl hydroxylase by minoxidil, as demonstrated to occur *in-vitro*, apparently did not occur in this *in-vivo* model. After the discovery of the inhibitory action on LH gene expression, minoxidil was postulated to possess anti-fibrotic properties by reducing the total number of hydroxylysine residues in the collagen molecule and consequently, the formation of hydroxyallysine cross-links (5, 9, 13). To achieve a reduction in hydroxyallysine crosslinking, only a decrease in the number of telopeptidehydroxylysine residues is required, but not a considerable reduction of the total number of hydroxylysine residues in the collagen molecule (16).

Moreover, minoxidil appeared to be able to decrease the LH mRNA level, but remarkably, no consistent decrease in the total number of pyridinoline cross-links was found in the matrix deposited by fibroblasts. Since minoxidil does not completely inhibit LH gene expression at a concentration of 500 µM, an explanation could be that residual gene expression is sufficient for hydroxylation of telopeptide lysine residues to a level of normally found in collagen produced by fibroblasts (16).

Our results demonstrated that minoxidil actively induced angiogenesis in burned area of the skin from first days of topical application. Minoxidil is either considered as a factor that acts directly on VEGF synthesis, or indirectly, by stimulating the synthesis of other cytokines or growth factors which themselves act directly on VEGF synthesis. It is reported that abundant VEGF production may ultimately lead to the formation of new blood vessels to maintain adequate microvascularization (7). 

We should also acknowledge the limitations of this study. Investigating pharmacokinetic and pharmacodynamic parameters including dose-response characteristics, bioavailability, absorption and metabolism parameters of minoxidil gel formulations could be more helpful for inferring the *in-vivo* results. Combination therapies and mixing treatment groups like animals receiving minoxidil formulations and SSD or dexpanthenol ointments could have resulted in more practical and applicable findings regarding burn wound healing. Nonetheless, such grouping patterns inevitably increased the number of experimental animals, which of course was not feasible in this study.

## Conclusion

In conclusion, although topical minoxidil improved the process of wound healing, our results did not support the proposed idea of minoxidil usage as an anti-fibrotic agent, because it did not alter the collagen content or tensile strength of burned area of the skin. However, in order to reject the possible effects of minoxidil as an anti-fibrotic agent, more objective animal models,* e.g.* hypertrophic scar model should be developed and studied. 
